# Exploring genetic diversity and phylogenetic connections of tropical bed bugs, *Cimex hemipterus* (F.) infestation in Indonesia

**DOI:** 10.1371/journal.pone.0327540

**Published:** 2025-07-09

**Authors:** Martini Martini, Retno Hestiningsih, Sri Yuliawati, Li Lim, Abdul Hafiz Ab Majid

**Affiliations:** 1 Laboratory of Health Entomology, Faculty of Public Health, Universitas Diponegoro, Semarang, Indonesia; 2 Department of Epidemiology and Tropical Disease Faculty of Public Health, Universitas Diponegoro, Semarang, Indonesia; 3 Household & Structural Urban Entomology Laboratory, School of Biological Sciences, Universiti Sains Malaysia, USM, Penang, Malaysia; Beni Suef University Faculty of Veterinary Medicine, EGYPT

## Abstract

Bed bugs pose significant public health challenges in tropical regions like Indonesia. This study aimed to identify the species of bed bugs involved in bed bug infestations across Indonesia using morphological and molecular approaches. Specimens were collected from 10 locations, including Central Java, Papua, and Kalimantan. A total of 101 *C. hemipterus* specimens were collected and examined morphologically, of which 23 were further analyzed using *COX1* gene sequencing. Morphological identification confirmed all samples as *C. hemipterus*, with pronotum width-to-length ratios consistent with established keys. Molecular analysis of *COX1* gene sequences revealed 98.61% to 99.77% similarity to reference sequences from Malaysia and Iraq. Phylogenetic analysis revealed strong genetic similarity among Southeast Asian populations, with minor regional variations and a distinct sub-branch for Iranian samples. The findings highlight the adaptability of *C. hemipterus* to diverse environments and its widespread prevalence in Indonesia. This study provides insight into genetic diversity and a foundation for future epidemiological studies.

## 1. Introduction

Bed bugs (*Cimex* spp.), commonly known as “kutu busuk” in Indonesia, are blood-feeding insects that present considerable public health issues and nuisance problems, much like mosquitoes. In local dialects, they are also called tinggi (Javanese), kepinding, or tumbila (Sundanese). Belonging to the genus *Cimex*, the two primary species of concern are *Cimex lectularius* and *Cimex hemipterus*. While *C. lectularius* is predominantly found in temperate regions, *C. hemipterus* thrives in tropical and subtropical climates, making Indonesia a prime habitat for this pest. These insects inhabit various spaces, such as mattresses, furniture, and dark, damp environments, where they find suitable conditions to proliferate [1].

The resurgence of bed bugs represents a perplexing issue in entomology. Long periods of absence followed by sudden reappearances in large numbers suggest multiple contributing factors. Globalization, with the increased movement of people and goods, has facilitated their spread across regions. Additionally, the development of resistance to insecticides, particularly pyrethroids, has significantly hindered control efforts [2–4].

Although bed bugs are not recognized as vectors of disease, their bites can cause considerable physical and psychological distress. Physical effects include allergic reactions, skin irritation, and in severe cases, anemia due to chronic blood loss, particularly in vulnerable groups such as children and the elderly [5,6]. The psychological impacts are equally concerning, with victims often experiencing anxiety, insomnia, and a reduced quality of life due to persistent fear of being bitten [7].

Historically, bed bugs were widespread in Indonesia, particularly in homes, movie theaters, hotels, and boarding houses, until the late 1970s. By the 1980s, their presence became more pronounced in dormitories, campuses, and residential areas. Over the past two decades, surveys have continued to document their prevalence, including studies conducted in Gebang Village, Sragen, Central Java (2010), Bogor Agricultural University dormitories (2010 and 2015), and tourist destinations in Manado and Sitaro, North Sulawesi [8].

In 2023, a preliminary survey in Purbayasa Village, revealed that 57% of surveyed homes were infested with bed bugs, with many residents neglecting preventive measures, such as airing mattresses and furniture. However, the locations of infestations have not been systematically documented, and the specific species responsible for these infestations remain unidentified [9].

Mitochondrial DNA, particularly the cytochrome c oxidase subunit I (*COX1*) gene, has proven to be a reliable molecular marker for species identification and phylogenetic analysis due to its maternal inheritance, high mutation rate, and lack of recombination. These characteristics make *COX1* highly informative for detecting genetic variation within and between closely related populations [10].

Recent molecular studies have advanced our understanding of bed bug population genetics and their symbiotic relationships. For instance, Chebbah et al. [11] analyzed the molecular characterization and diversity of *Wolbachia* endosymbionts in bed bugs collected in Paris, highlighting the genetic complexity and symbiotic diversity within *Cimex* populations. Similarly, Djouaher et al. [12] provided the first official report of *Cimex hemipterus* infestations in Algeria, emphasizing the expanding geographic range of this species and the need for regional surveillance. These findings underscore the importance of molecular tools in revealing hidden diversity and tracking the spread of infestations.

Therefore, this study aims to identify the species responsible for bed bug infestations across multiple regions of Indonesia using both morphological and molecular characteristics. Additionally, phylogenetic analysis will be conducted to explore the diversity and genetic variations of bed bugs, providing insights into their distribution and evolutionary relationships.

## 2. Materials and methods

### 2.1. Bed bugs collection

**Fig 1:**
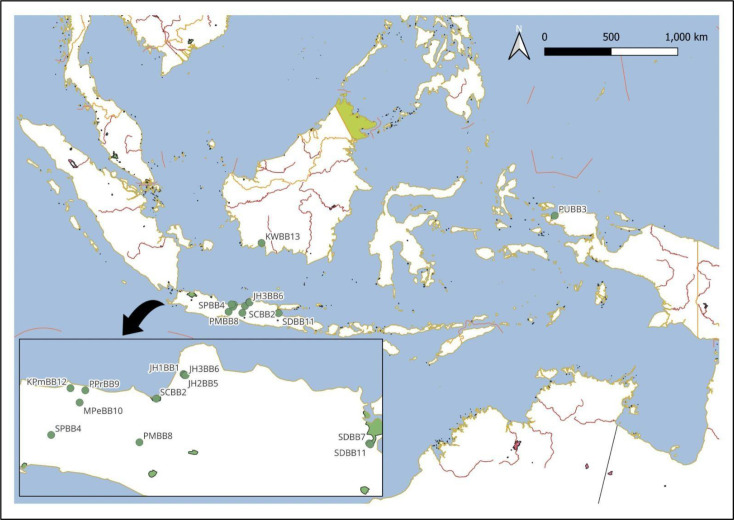
The locations for bed bugs collection. The abbreviation corresponds to Table 2. The map is sourced from Natural Earth (public domain): http://www.naturalearthdata.com/.

The bed bug specimens were primarily collected between September 2024 and January 2025 in Central Java Province, focusing on residential buildings such as houses, settlements, dormitories, orphanages, and Islamic boarding schools (pondok pesantren). Additional specimens were obtained from Papua and Kalimantan through colleague networks, resulting in a total of 10 collection sites across 9 regions in Indonesia (Fig 1). Field collections were conducted with permission from the Faculty of Public Health, Universitas Diponegoro (Ethical Approve number: 19/EA/KEPK-FKM/2025). All necessary permits were obtained prior to sampling, and access to each site was approved by local authorities and property owners. The latitude and longitude of each location are listed in Table 1, along with a code assigned to each site. All specimens were handpicked upon discovery from human dwellings. No traps or chemical agents were used during collection.

**Table 1:** Summary of bed bug sample coding from each collection location, including latitude and longitude coordinates, percentage similarity with reference sequences (determined via BLAST), corresponding GenBank accession numbers, and species identification.

Location	Number of samples	Latitude	Longitude	Code for sample selected for molecular ID	Species assigned	BLAST (%)	BLAST (accession number)
Jepara H1	6	N110°42’38.61’‘	W6°43’51.99’‘	JH1BB1	*C. hemipterus*	98.83	ON989839.1
Semarang	6	N110°25’22.01’‘	W6°59’30.48’‘	SCBB2	*C. hemipterus*	99.77	ON989839.1
Papua	1	N131°19’34.31’‘	W0°53’33.72’‘	PUBB3	*C. hemipterus*	99.77	ON989839.1
Purbalingga	6	N109°18’40.23’‘	W7°22’33.12’‘	SPBB4	*C. hemipterus*	99.53	KT851505.1
Jepara H2	9	N110°42’38.61’‘	W6°43’51.99’‘	JH2BB5	*C. hemipterus*	98.61	ON989832.1
Jepara H3	13	N110°42’38.61’‘	W6°43’51.99’‘	JH3BB6	*C. hemipterus*	99.54	ON989832.1
Sidoarjo	10	N112°40’34.86’‘	W7°27’52.01’‘	SDBB7	*C. hemipterus*	98.61	ON989832.1
Pondok magelang	10	N110°14’42.28’‘	W7°27’9.94’‘	PMBB8	*C. hemipterus*	99.31	KT851505.1
Darma Bakti Medona, Kota PKLN	10	N109°40’20.65’‘	W6°54’9.94’‘	PPrBB9	*C. hemipterus*	99.30	KT851520.1
Pekalongan, Jawa Tengah	8	N109°36’43.45’‘	W7°1’56.00’‘	MPeBB10	*C. hemipterus*	99.08	KT851520.1
Sidoarjo, Jawa Timur	9	N112°40’34.86’‘	W7°27’52.01’‘	SDBB11	*C. hemipterus*	99.08	KT851520.1
Pemalang Jawa Tengah	11	N109°30’54.94’‘	W6°52’49.63’‘	KPmBB12	*C. hemipterus*	99.31	KT851520.1
Kotawaringin	2	N111°32’1.38’‘	W2°43’51.50’‘	KWBB13	*C. hemipterus*	99.07	KT851505.1

**Table 2:** *Cimex* sequences used in phylogenetic analysis.

Species	Code	State/Country	Reference	GenBank accession number
*C. adjunctus*	HEM303509	Canada	[13]	KR035747
*C. lectularius*	CR2017	Costa Rica	[14]	MN271345
*C. hemipterus*	IPP SNA003	Malaysia: Perak	[15]	KT851505
*C. hemipterus*	GEPP SNA018	Malaysia: Penang	[15]	KT851520
*C. hemipterus*	IBKADB	Iraq	[16]	ON989833.1
*C. hemipterus*	IEKOY	Iraq	[16]	ON989839.1
*C. hemipterus*	agri1	Thailand	[17]	JX826468
*C. hemipterus*	gate2	Thailand	[17]	JX826469
*C. hemipterus*	sarin3	Thailand	[17]	JX826470
*C. hemipterus*	Az	Iran	[18]	MG739320
*C. hemipterus*	Hm	Iran	[18]	MG739319
*C. hemipterus*	Tn	Iran	[18]	MG739321
*C. hemipterus*	KPmBB12	Indonesia: Central Java	Present study	PQ731951
*C. hemipterus*	KWBB13	Indonesia: Kotawaringin	Present study	PQ731952
*C. hemipterus*	MPeBB10	Indonesia: Central Java	Present study	PQ731953
*C. hemipterus*	PPrBB9	Indonesia: Central Java	Present study	PQ731954
*C. hemipterus*	SDBB11	Indonesia: Sidoarjo	Present study	PQ731955
*C. hemipterus*	JH2BB5	Indonesia: Jepara	Present study	PQ731956
*C. hemipterus*	JH3BB6	Indonesia: Jepara	Present study	PQ731957
*C. hemipterus*	PMBB8	Indonesia: Magelang	Present study	PQ731958
*C. hemipterus*	SDBB7	Indonesia: Sidoarjo	Present study	PQ731959
*C. hemipterus*	JH1BB1	Indonesia: Jepara	Present study	PQ731960
*C. hemipterus*	PUBB3	Indonesia: Papua	Present study	PQ731961
*C. hemipterus*	SCBB2	Indonesia: Semarang	Present study	PQ731962
*C. hemipterus*	SPBB4	Indonesia: Purbalingga	Present study	PQ731963

### 2.2. Morphological identification

All the bed bug specimens collected at each location were examined under a dissecting microscope (Olympus, SZ-LGR66, Japan). The pronotum’s width-to-height ratio was measured following the method described by Usinger [19].

### 2.3. DNA extraction, PCR amplification, and sequencing

**Fig 2:**
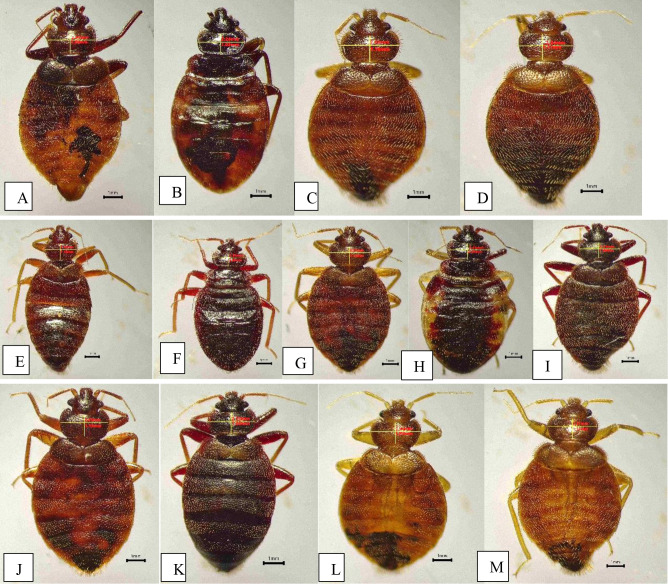
Samples that were randomly selected from each collection site for DNA extraction. A: Sample JH1BB1. B: Sample SCBB2. C: Sample PUBB3. D: Sample SPBB4. E: Sample JH2BB5. F: Sample JH3BB6. G: Sample SDBB7. H: Sample PMBB8. I: Sample PPrBB9. J: Sample MPeBB10. K: Sample SDBB11. L: Sample KPmBB12. M: Sample KWBB13.

One specimen was randomly selected from each collection site for DNA extraction (Fig 2, Table 1). Each bed bug sample was sterilized using 70% ethanol and rinsed with sterile distilled water. DNA was extracted using the HiYield™ Genomic DNA Isolation Kit (Real Biotech Corporation, Taiwan) according to the manufacturer’s protocol. The mitochondrial cytochrome c oxidase subunit I (*COX1*) gene was targeted to identify the bed bug species. Amplification of the *COX1* gene was carried out using the primers CHP10-F (5’-TTCGGAATGTGGGCAGGGAT-3’) and CHP10-R (5’-GGTTATTCCGGCAGGACGTAT-3’) as described by Seri Masran and Ab Majid [19].

The PCR was performed with a Thermal Cycler (TaKaRa, Japan) in a 25 μL reaction volume, comprising 12.5 μL of 2 × Green Taq PCR Mastermix (NX, Malaysia), 0.5 μL of 10 mM forward and reverse primers, 5 μL of DNA template, and sterile cold distilled water to reach the final volume. The PCR program included 40 cycles of denaturation at 94°C for 30 seconds, annealing at 50°C for 30 seconds, extension at 75°C for 45 seconds, and a final extension at 75°C for 5 minutes.

The PCR products were visualized on a 1.0% agarose gel and were purified using the PrimeWay Gel Extraction/PCR Purification Kit (First Base, Malaysia). The purified products were then sent to First Base Laboratories Sdn. Bhd. (Malaysia) for sequencing.

### 2.4. Species assignment and phylogenetic analysis

The *COX1* sequences were analyzed using BLAST against the NCBI database. Each sample’s identity was determined based on the closest match of the acquired sequences to the GenBank query sequences. The sequences obtained in this study were deposited in GenBank with accession numbers PQ731951–PQ731963 (Table 2). The same sequences are also available in the Figshare repository with https://doi.org/10.6084/m9.figshare.29162066.

Multiple sequences, including the samples, reference sequences, and outgroups, were aligned using Clustal W in the Molecular Evolutionary Genetics Analysis (MEGA7) (version 7) software [20] and manually edited. Reference sequences (*C. hemipterus* sequences from Iran, Thailand, Malaysia, and Iraq) and outgroups (*C. adjunctus* and *C. lectularius*) used for the phylogenetic analysis were downloaded from NCBI GenBank and were listed in Table 2. Outgroup sequences were selected from closely related Cimicidae species based on their taxonomic proximity to *C. hemipterus* and the availability of high-quality, complete *COX1* sequences in GenBank. A model test was conducted to identify the best-fit DNA substitution model for constructing a Maximum Likelihood (ML) phylogenetic tree. The model with the lowest Akaike Information Criterion (AIC) score was selected for the ML tree construction. The stability and reliability of the phylogenetic tree were evaluated using bootstrap analysis with 1,000 replications.

### 2.5. Genetic diversity of *COX1* sequences

DNA Sequence Polymorphism (DNAsp) (v6.12.03) [21] was used to analyze the *COX1* sequences of bed bug samples collected from various locations in Indonesia. The analysis included determining the number of haplotypes, haplotype diversity, nucleotide diversity, segregating sites, mutations, and estimates of Theta-W and Tajima’s D.

### 2.6. Population genetic structure through *COX1* sequences

The population genetic structure of bed bugs between Indonesia and those from other countries, including Iran, Thailand, and Malaysia (sequences downloaded from NCBI database) (Table 2), was assessed using pairwise genetic distance (*F*_*St*_) through DNAsp (v6.12.03) [21]. Statistical significance was determined through 1,000 permutations.

## 3. Results

### 3.1. Morphological identification

The mean (±SD) pronotum width-to-length ratio of the collected bed bugs was approximately 2.281 ± 0.175 mm (Fig 2). These results align with the identification keys provided by Usinger [19], which state that the pronotum width-to-length ratio in *Cimex hemipterus* is less than 2.5 mm. Consequently, the collected bed bug samples were tentatively identified as *C. hemipterus*.

### 3.2. Species assignment and phylogenetic analysis of bed bugs through *COX1* sequences

All sequences of bed bugs displayed 98.61–99.77% similarity with *C. hemipterus* (ON989839.1, ON989832.1, KT851505.1, KT851520.1) (Table 1) specimens from Malaysia and Iraq (Table 2).

**Fig 3:**
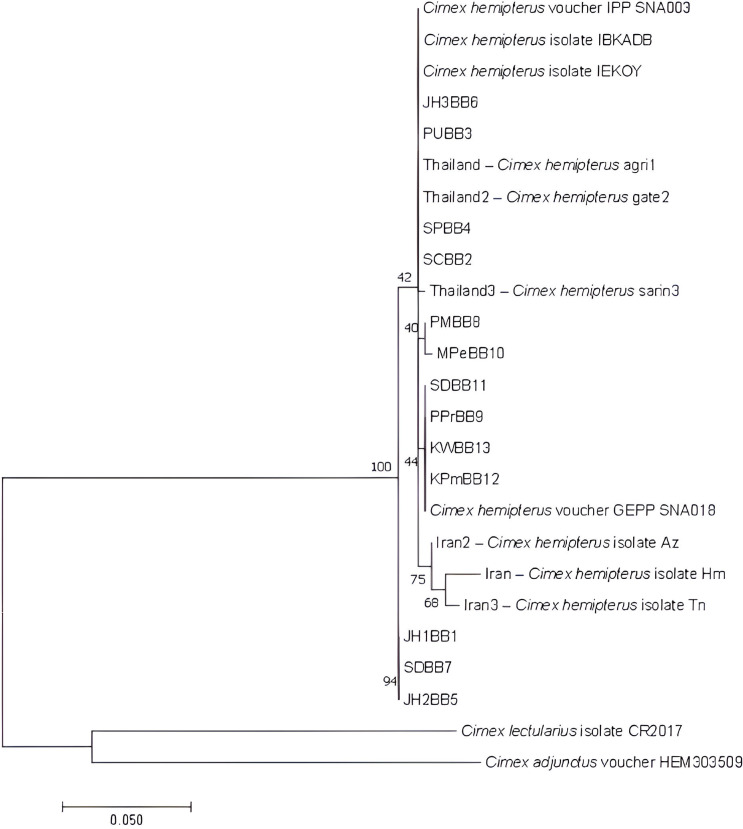
Maximum likelihood phylogenetic tree based on *COX1* sequences. *Cimex lectularius* CR2017 and *C. adjunctus* HEM303509 are the outgroups. Abbreviation refers to Tables 1 and 2.

The phylogenetic tree constructed using the Maximum Likelihood (ML) method with the Tamura 3-parameter model (T92 + G) is presented in Fig 3. All *Cimex hemipterus* sequences are grouped into a single clade with several sub-branches. Consistent with the BLAST results, the sequences from this study showed high similarity to sequences from Iraq and Malaysia and were naturally clustered together in the phylogenetic analysis, along with sequences from Thailand. In contrast, sequences from Iran formed a distinct sub-branch within the tree.

### 3.3. Genetic diversity analysis of *COX1* sequences

Among all *COX1* sequences from Indonesia, six haplotypes were identified. The overall haplotype diversity (Hd) was 0.846 ± 0.065, while nucleotide diversity was low (Pi = 0.00578) (Table 3).

**Table 3:** Genetic diversity of bed bugs sequences from Indonesia.

Population	Indonesia
n	13
No. haplotypes	6
Haplotype diversity (Hd)±SD	0.846 ± 0.065
Nucleotide diversity (Pi)	0.006
No. segregating sites	6
Total no. mutations (Eta)	6
Theta (per sequence) from S, Theta-W	1.933
Tajima’s D	0.568 (*P > 0.10)

* P > 0.01 indicated no significant difference.

### 3.4. Population genetic structure

Indonesia’s population showed the smallest genetic distance from Malaysia’s population while having the greatest genetic divergence from Iran’s population (Table 4).

**Table 4:** DNA divergence between populations.

Population	Number of nucleotide differences, k	Nucleotide diversity, Pi(t)	H_s_	*K*_s_	*F*_*St*_
Indo vs Iran	3.650	0.009	0.8590	2.938	0.348
Indo vs Thailand	1.992	0.005	0.8312	1.938	0.103
Indo vs Malaysia	1.941	0.005	0.8442	1.941	0.000

## 4. Discussion

The morphological identification of bed bugs collected in this study revealed that all specimens exhibited pronotum width-to-length ratios consistent with the diagnostic keys for *C. hemipterus* as described by Usinger [19]. Molecular analysis using *COX1* gene sequences further confirmed the species designation, with all samples displaying a high similarity (98.61–99.77%) to *C. hemipterus* sequences in the GenBank database, including those from Malaysia (ON989839.1, KT851505.1) and Iraq (ON989832.1) (Table 1). The observed similarity range of 98.61% to 99.77% in *COX1* sequences is consistent with typical intraspecific variation reported for insects, including bed bugs. In most insect species, *COX1* sequence similarities above 98% generally indicate conspecific individuals, with variation arising due to geographic isolation or population structure [22,23]. Prior studies on *C. hemipterus* have also reported similar levels of intraspecific *COX1* variation across different geographic regions [15,24], further supporting the identification of all collected samples as *C. hemipterus*. These results also align with prior studies in Southeast Asia [24,25], affirming the dominance of *C. hemipterus* in tropical and subtropical climates. The use of both morphological and molecular methods strengthens the reliability of these findings, highlighting the importance of integrating diverse approaches in taxonomic studies.

The genetic diversity analysis identified six haplotypes within the Indonesia *COX1* sequences, with an overall haplotype diversity (Hd) of 0.846 ± 0.065, indicating a moderately high level of genetic variation (Table 3). However, nucleotide diversity was low (n = 0.006), suggesting that the genetic differences between the haplotypes are relatively small. The presence of only six segregating sites and mutations further supports the conclusion of limited genetic variation within the Indonesia *C. hemipterus* population. High haplotype diversity and low nucleotide diversity are often observed in populations that have recently expanded [26]. During population growth, new haplotypes may emerge through mutations or migrations, but there hasn’t been enough time for significant nucleotide differences to accumulate between them. This pattern is commonly seen in species with rapid dispersal or in populations recovering from a bottleneck [27], such as bed bugs, which can easily hitchhike on human belongings and disperse and have also suffered from bottlenecks [24]. The Tajima’s D value of 0.5678 (P > 0.10) indicates no significant departure from neutrality, suggesting a lack of strong selection pressures acting on the population and a potential equilibrium state for the bed bug populations in Indonesia. These findings highlight the adaptability of *C. hemipterus* to local environmental conditions while maintaining a stable genetic structure.

The analysis of DNA divergence between populations provides additional insights into the genetic relationships of *C. hemipterus* across regions (Table 4). Indonesia’s population showed the smallest genetic distance to Malaysia’s population, with a mean number of nucleotide differences (k) of 1.941 and an *F*_*St*_ value of 0.000, indicating virtually no genetic differentiation. This close genetic relationship is consistent with the geographical proximity and shared ecological conditions between Indonesia and Malaysia. In contrast, comparisons with Iran’s populations revealed the greatest genetic divergence, with the highest *F*_*St*_ value (0.348) and the largest number of nucleotide differences (k = 3.650). This divergence likely reflects regional genetic isolation and adaptations to differing environmental conditions. Comparisons with Thailand’s population indicated moderate differentiation, with an *F*_*St*_ of 0.10317 and k = 1.992, suggesting some shared evolutionary history but also localized genetic variation.

The phylogenetic analysis (Fig 3) further supports these observations, as Indonesia *C. hemipterus* sequences clustered closely with those from Malaysia, Thailand, and Iraq, reflecting their shared evolutionary history [28]. However, Iran sequences formed a distinct sub-branch, emphasizing regional genetic differentiation within *C. hemipterus*. These findings suggest that while the population from Indonesia maintains close genetic ties with neighboring Southeast Asia populations, geographic and environmental factors have led to subtle but notable genetic divergence in more distant regions such as Iran [29].

Despite the wide geographic spread of the collection sites (from Central Java to Papua and Kalimantan), *C. hemipterus* populations in Indonesia exhibited limited genetic divergence, forming a relatively uniform subclade (Fig 3). This suggests that *C. hemipterus* has a high capacity for ecological adaptation, enabling it to establish stable populations across diverse environmental conditions and urban settings. The ability of this species to thrive in both densely populated urban areas and more remote locations supports its classification as a highly adaptable and resilient pest [4,30–33]. Moreover, the relatively high similarity between *C. hemipterus* samples from geographically distant regions may reflect human-mediated dispersal, particularly through increased domestic travel and movement of infested belongings. Bed bugs are known to spread via passive transport, often hitchhiking on luggage, furniture, or clothing [34,35]. This pattern of high genetic similarity across distant locations has also been observed in other studies [19,36]. It supports the idea that human activity plays a major role in shaping the genetic structure of bed bug populations.

Although bed bugs are not known to transmit diseases, their bites can cause considerable physical and psychological distress. Physical effects include allergic reactions, skin irritation, and, in severe cases, anemia due to chronic blood loss. Psychological impacts, such as anxiety and insomnia, are equally significant and can substantially reduce the quality of life for affected individuals [5,6]. The high prevalence of infestations documented in this study underscores the urgent need for public health interventions, including awareness campaigns to educate communities about preventive measures and early detection.

Based on the findings, several recommendations can be made to address the resurgence of bed bugs in Indonesia. First, targeted awareness campaigns should emphasize the importance of preventive practices, such as regular cleaning and airing of bedding and furniture [35]. Second, integrated pest management strategies should be developed, combining chemical control with non-chemical methods such as heat treatments and environmental modifications [37]. Third, the genetic analysis of this study reveals low differentiation among populations across geographically distant regions, suggesting passive, human-mediated dispersal. This insight highlights the need for coordinated region-wide control efforts rather than isolated interventions. Monitoring high-risk areas such as transportation hubs, hotels, and secondhand markets can help prevent further spread. Finally, continued monitoring of genetic variations and insecticide resistance is essential to inform the development of effective, region-specific control measures.

## 5. Conclusion

In this study, morphological characterization and molecular analysis confirmed that all bed bug species found in Indonesia belong to *C. hemipterus*. Their *COX1* gene sequences showed 98.61% to 99.77% similarity to reference sequences from Malaysia and Iraq. These findings provide valuable insights and a foundation for addressing the resurgence of bed bugs in Indonesia and beyond.
